# Exploring career interest and STEM self-efficacy: implications for promoting gender equity

**DOI:** 10.3389/fpsyg.2024.1402933

**Published:** 2024-10-11

**Authors:** Niam Yamani, Hiya Almazroa

**Affiliations:** ^1^Learning & Outreach Director Diriyah Foundation, Riyadh, Saudi Arabia; ^2^Teaching and Learning Department, College of Education and Human Development, Princess Nourah bint Abdulrahman University (PNU), Riyadh, Saudi Arabia

**Keywords:** STEM, career interest, self-efficacy, gender and STEM, Saudi Arabia STEM, female students, Saudi Arabia

## Abstract

This study explores the relationship between career interests and self-efficacy in Science, Technology, Engineering, and Mathematics (STEM) among young girls in Riyadh, Saudi Arabia. Employing a quantitative research design with a correlational approach, we utilize the S-STEM survey to measure changes in students’ STEM subject efficacy and their interest in STEM careers. Our sample comprises 671 middle and high school female students from 49 schools, representing a diverse cross-section of the population. The findings indicate that female students exhibit high levels of STEM self-efficacy and have a strong preference for medicine as a career choice. While the overall interest in STEM careers among students is moderate, there are notable variations in interest across different STEM fields. Importantly, a positive and significant correlation was observed between female students’ interest in STEM careers and their self-efficacy in STEM. This research holds important implications for the promotion of STEM education and careers among female students. By recognizing the unique context of Saudi Arabia and the perspectives of young girls in Riyadh, this study offers a fresh perspective on the factors influencing STEM career aspirations and highlights the importance of fostering self-efficacy beliefs among female students.

## 1 Introduction

With many countries striving to bolster enrollment in Science, Technology, Engineering, and Mathematics (STEM) fields and to encourage greater participation of women and minorities in STEM education to meet the growing demand for STEM careers (Popa and Ciascai, 2017), the persistent underrepresentation of these groups in STEM disciplines remains a global issue. Studies, like that of Luo et al. (2021), indicate that female students with elevated levels of self-efficacy in STEM are more inclined toward pursuing careers in STEM fields. Self-efficacy, characterized as an individual’s belief in their capacity to successfully accomplish tasks, is closely associated with career aspirations and opportunities (Lent et al., 2011). Notably, low self-efficacy stands out as a primary deterrent for women and minorities considering careers in STEM (Gremillion et al., 2019). Research findings highlight that individuals with robust STEM self-efficacy are more likely to enroll in STEM programs, persist for longer durations, and achieve superior outcomes compared to those with lower levels of STEM self-efficacy (Falco and Summers, 2017).

Social Cognitive Career Theory (SCCT) suggests that improving self-efficacy in STEM fields and developing positive and realistic expectations can lead to STEM career interest (Mau et al., 2020). Self-efficacy is an important variable in contributing to SCCT and is connected to career goals and options (Lent et al., 2011). SCCT explains how individuals use self-efficacy to make career choices and examines the mechanisms through which career interests develop (Lent et al., 1994). It encompasses elements such as self-efficacy, goals, expectations, and social-cultural factors (Yusoff et al., 2019). The integration of student attitudes and career interests in the SCCT model indicates that self-efficacy levels predict students’ intent to enter STEM careers (Lent et al., 2000). Self-efficacy has been identified as a key variable in SCCT, influencing career choices, goals, and options (Turner et al., 2003). Applying SCCT to female students can result in higher STEM self-efficacy and increased interest in STEM careers (Luo et al., 2021). SCCT provides guidance for female students in choosing and developing careers, helping them overcome challenges they may encounter in pursuing STEM careers (Blotnicky et al., 2018). By utilizing SCCT, female students can navigate their career paths and increase their chances of success in STEM fields.

Most of the work that investigates gender differences in STEM pursuit has captured the experiences and perceptions of individuals from Western, and Industrialized countries, where women are underrepresented in STEM (Folberg and Kaboli-Nejad, 2020). In some Muslim-majority and Middle Eastern countries women are overrepresented in STEM. UNESCO (2019) reported that women and men are approximately equally represented in STEM in Arab states, Central Asia, and Latin America. Therefore, exploring the relationship between STEM career and STEM self-efficacy specifically among Saudi female students would provide valuable insights into a unique cultural context where women are overrepresented in STEM.

Moreover, it has been discovered that the cultural context plays a significant role in shaping perceptions and attitudes toward STEM fields (Brown et al., 2017). Considering the cultural disparities between Western countries and Saudi Arabia, it becomes crucial to investigate how cultural factors may influence the perceptions of female Saudi students regarding STEM careers. Understanding the impact of culture on perceptions of STEM is essential for comprehending the disparities in STEM pursuit based on gender and ethnic groups (Folberg and Kaboli-Nejad, 2020). According to UNESCO (2017), there are multiple factors at various levels—individual, family, institutional, and societal—that influence women’s participation, achievement, and progression in STEM studies and careers.

Furthermore, the existing literature highlights a significant research gap in the study of STEM pursuit in the Arab world (Fadlelmula et al., 2022), including Saudi Arabia (Alghamdy and Almazroa, 2024). Limited research has specifically examined gender differences in STEM pursuit among individuals in non-Western nations (Brown et al., 2017). Therefore, conducting a study that focuses on Saudi female students would contribute to filling this gap in knowledge and provide insights into the factors influencing their STEM career interest. By investigating the relationship between career interest and STEM self-efficacy among Saudi female students, we aim to contribute to understanding the factors influencing STEM pursuit in a non-Western, predominantly Muslim-majority country where women are overrepresented in STEM (Folberg and Kaboli-Nejad, 2020; UNESCO, 2019; Fadlelmula et al., 2022). This research will contribute to a more comprehensive understanding of the factors influencing STEM career interest among female students and inform efforts to promote and support their participation in STEM fields.

The significance of this study lies in its potential to shed light on the factors influencing female students’ interest in pursuing STEM careers. Understanding the level of self-efficacy and career interest among female students is crucial for educational policymakers and stakeholders in developing targeted interventions and strategies to promote and support their engagement in STEM fields. Furthermore, this study addresses the influence of cultural and educational factors on female students’ interest in STEM. Saudi Arabia has its unique cultural and gender segregated educational context, and exploring the relationship between career interest and STEM self-efficacy within this context can provide valuable insights. The findings of this study may contribute to the existing body of knowledge on gender diversity and inclusivity in STEM education and career pathways (Folberg and Kaboli-Nejad, 2020).

### 1.1 Research hypothesis

The Null Hypothesis: there is no significant correlation between the STEM careers and the corresponding fields of STEM. In other words, the correlation coefficients are equal to zero.

The alternative Hypothesis: there is a significant correlation between the STEM careers and the respective fields of STEM. The correlation coefficients are not equal to zero, indicating a positive relationship between the variables.

### 1.2 Research question

This study seeks to explore the levels of STEM career interest and STEM self-efficacy among middle and high school female students in Saudi Arabia. The research aims to address the following inquiries:

1. What is the level of STEM career interest among Saudi female students?
2. What is the level of STEM self-efficacy among Saudi female students?
3. Is there a correlation between STEM career interest and STEM self-efficacy among Saudi female students?

### 1.3 Review of literature

#### 1.3.1 STEM career interests

The significance of STEM professions spans a wide range of fields, playing a pivotal role in driving innovation, addressing global challenges, and contributing to economic growth across industries. Recognizing the link between a country’s economic strength and advancements in science and technology, the National Academy of Sciences emphasizes the importance of STEM fields (National Academy of Sciences, National Academy of Engineering, and Institute of Medicine, 2007). However, despite the increasing importance of STEM, there remains a disparity in the representation of females and minorities in these fields (Fouad and Santana, 2016). Numerous factors have been identified as contributors to the underrepresentation of females in STEM. Su et al. (2009) observed that women tend to show more interest in careers involving working with people, while men are more interested in careers involving working with objects. Considering that many STEM careers involve object-oriented work, this could contribute to the observed gender disparities. Also, Wang and Degol (2016) found that females are less represented in STEM fields that require intensive mathematical skills. This underrepresentation can be influenced by various factors, including cognitive abilities, occupational preferences, work-family balance, field-specific ability beliefs, gender-related stereotypes, and workplace bias.

Exposure to female experts in STEM fields has been shown to have a positive impact on the attitudes and career aspirations of female students toward pursuing STEM careers (Stout et al., 2011). In a separate study by Zhou et al. (2021) focusing on primary students, comparisons were made regarding their career interests in math, science, and engineering/technology. The results indicated a higher interest in engineering/technology, lower mean scores in math and science domains, and a higher mean score in the engineering/technology domain. Ramsey emphasizes the importance of agentic traits in achieving success in STEM careers, highlighting their greater value compared to communal traits. Negative stereotypes surrounding science and mathematics can deter female adolescents from pursuing STEM careers (Ramsey, 2017). STEM professions are often viewed as conflicting with communal objectives, and women’s inclination toward communal goals is inversely related to their interest in STEM careers (2017). Another significant factor is the educational attainment of parents, which positively correlates with pursuing higher education and STEM careers, while the impact of socioeconomic background appears to be less pronounced (Chachashvili-Bolotin et al., 2016). Stout et al. (2011) further support the idea that exposure to female experts in STEM fields positively influences the attitudes of female students toward the STEM field and boosts their aspirations to pursue careers in STEM.

The review of existing literature underscores the importance of student attitudes and STEM self-efficacy in predicting their inclination toward pursuing careers in STEM fields. Numerous studies have illustrated how attitudes and interests significantly influence students’ career decisions, particularly in subjects like science and mathematics, shaping their interest in STEM careers across various educational stages (Gremillion et al., 2019; Razali et al., 2018). Research has delved into the role of self-efficacy in STEM and its impact on career choices, taking into account variables such as gender and school types. An extensive survey conducted by Wiebe et al. (2013) involving 15,000 students explored the interplay between STEM careers, attitudes toward STEM subjects, and interest in STEM careers at different academic levels. The findings revealed that students generally hold moderately positive attitudes toward science, mathematics, engineering, and technology. However, female students exhibited lower levels of interest in specific STEM career paths compared to their male peers, who demonstrated comparable interest levels across all STEM disciplines. In a study by Chan (2022), the relationship between career interest and STEM interest was investigated, uncovering a positive correlation between the two variables. Additionally, a stronger sense of self-efficacy was associated with heightened interest in pursuing careers in STEM fields.

#### 1.3.2 STEM self-efficacy

Self-efficacy, as described by Bandura (1977), refers to people’s assessments of their own capacities to plan and carry out actions necessary for achieving specific types of performances. It represents an individual’s confidence in their ability to exert control over their behavior and circumstances that impact their lives. Self-efficacy beliefs are fundamental to individuals’ functioning, and confidence in successfully carrying out necessary behaviors is crucial in both common and challenging situations (Bandura, 1997). Research evidence suggests that students with strong self-efficacy in various academic disciplines are more likely to engage in activities that facilitate the growth of their knowledge, abilities, and skills in those areas. They demonstrate persistence in the face of challenges and exhibit a willingness to tackle difficult tasks for an extended period (Artino et al., 2010). Furthermore, students’ conceptions of competence, or their assumptions about self-efficacy evaluation, have been found to predict their motivation and subsequent educational choices more strongly than actual skill (Stone, 2000). Higher levels of self-efficacy lead to “approach” behaviors, wherein individuals are inclined to gravitate toward tasks they believe they can successfully accomplish. In contrast, “avoidance” behaviors are associated with lower self-efficacy (Betz and Hackett, 2006; Ng and Lovibond, 2020).

Consequently, students with high self-efficacy in STEM fields are more likely to pursue STEM careers. The word STEM was originated by the National Science Foundation in the early 2000s as an acronym to a set of disciplines: Science, Technology, Engineering, and Math (Donahoe, 2013). Bandura (2006) and others have recommended that teachers employ instructional strategies that not only promote students’ acquisition of educational skills and knowledge but also encourage the growth of necessary supplementary confidence. STEM education plays a crucial role in preparing students for future careers in science, technology, engineering, and mathematics. Understanding students’ attitudes and interest in STEM fields is essential for promoting their engagement and participation in these disciplines. Ibrahim and Şeker (2022) conducted a study examining middle school students’ attitudes toward STEM education in Turkey and Ghana. Their findings revealed overall positive attitudes toward STEM education among students. However, they identified significant effects of gender and school location on attitudes toward engineering, highlighting the importance of considering gender-related factors when investigating students’ interest in STEM careers.

Research conducted by Chan (2022) for secondary schools in China, indicated that girls had lower self-efficacy in STEM, perceiving themselves as less competent to perform well in STEM tasks compared to boys. This finding underscores the need to address gender-related factors when examining students’ self-efficacy and career interest in STEM. Bartholomew and Santana (2021) explored the perceptions of primary-school children in the US regarding STEM fields before and after an eight-week STEM literature intervention. The study revealed that exposure to STEM-focused literature had a positive impact on students’ perceptions of STEM, particularly among female students. This highlights the potential of interventions, such as exposure to STEM literature, in shaping students’ attitudes and interest in STEM.

Kucuk and Sisman (2020) investigated the influence of robotics activities on secondary school students’ attitudes toward robotics and STEM in Turkey. While their study primarily focused on robotics, it provided insights into gender differences in desire and confidence for robotics learning. These findings emphasize the importance of considering gender-related factors when examining students’ self-efficacy and career interest in STEM. Ching et al. (2019) evaluated the effects of a project-based STEM integrated robotics curriculum on elementary school students’ attitudes toward STEM in the US. Their study found a significant increase in students’ attitudes toward math, which was attributed to their engagement in building and programming robots and utilizing math in problem-solving. This suggests that hands-on, project-based learning experiences can positively influence students’ attitudes toward STEM subjects. Furthermore, Ladeji-Osias et al. (2016) focused on the Summer Minority Male Maker Program and its impact on middle school boys’ interest in STEM careers. The program involved instruction and mentoring by college students, resulting in increased interest in STEM subjects, design careers, and entrepreneurial skills among the participants. Although the study targeted male students, it highlights the potential impact of targeted programs and mentorship opportunities on students’ interest in STEM.

Based on the literature review conducted, it is evident that established issues regarding low STEM efficacy among females and the underrepresentation of women in STEM careers exist in the Western context. However, the available research does not align with these findings in the Middle East. In fact, there is a growing popularity of STEM careers in the region, with more women than men graduating in fields such as biology, computer technology, mathematics, statistics, and physics in countries like Saudi Arabia. This indicates a different trend compared to the findings in Western literature (Islam M. M., 2019; Islam S. I., 2019; Saudi Women Making Their Mark in Science, 2021).

While the reviewed literature provides valuable insights into the factors influencing student attitudes, career interests, and STEM self-efficacy, there is a relative lack of studies addressing STEM in the Arab world. Further research is needed, particularly in the Arab context, to gain a more comprehensive understanding of the dynamics influencing students’ interest in STEM fields in different regions. Conducting additional studies in the Middle Eastern context would allow for the identification of factors specific to the region and the development of targeted strategies to promote and support women’s participation in STEM fields.

Overall, the literature indicates the need to foster positive attitudes and interest in STEM fields, particularly among female students. Building upon the existing knowledge, this study aims to provide valuable insights into the factors influencing female students’ self-efficacy and career interest in STEM in Saudi Arabia, where the underrepresentation of women in STEM fields has been attributed to low STEM-career self-efficacy, a lack of self-confidence in STEM subjects, and a scarcity of social support and encouragement to pursue STEM-related educational and career goals (Falco and Summers, 2017).

#### 1.3.3 Context of the study

The Saudi government has prioritized reforming the status of women (Saudi Vision 2030, 2016). Women constitute 58% of Saudi university graduates, and increasing their participation in the workforce is a recognized government priority. However, the employment rate of females with a bachelor’s degree stands at only 62%, however, the Saudi Vision aims to raise female workforce participation to 30% by 2030, and the country has shown promising progress in gender parity, reporting the highest increase among 14 countries (World Economic Forum, 2020). In terms of STEM education, women in Saudi Arabia have surpassed men in graduating with a bachelor’s degree in fields such as biology, computer technology, mathematics, statistics, and physics (Saudi Women Making Their Mark in Science, 2021). It is widely recognized that a country’s economic strength is linked to advancements in science and technology. However, there is still a significant underrepresentation of women and minorities in STEM fields (Fouad and Santana, 2016).

## 2 Methodology

### 2.1 Research design

A quantitative research design using a survey method was chosen for this study to determine the relationship between career interests and STEM self-efficacy among students. Quantitative research is rooted in a positivist paradigm, focusing on measuring independent facts within a tangible reality (Healy and Perry, 2000). This enables researchers to maintain a rational and unbiased interest in studying social systems (Denscombe, 2017). The quantitative method employed within this paradigm focuses on statistical information and quantifiable factors, which hold significance in the real world. Moreover, data collection is conducted under controlled conditions to minimize confounding variables and ensure that the observed positive associations are attributed to the variables of interest (Park and Park, 2016). In this study, both inductive and deductive theories are utilized. The inductive approach will emphasize exploring STEM self-efficacy and career interests, while the deductive theory will examine the relationships between these factors, a quantitative research technique is deemed appropriate for this study. In this case, the study seeks to determine the relationship between STEM self-efficacy and career interests among students.

### 2.2 Participants

The present study focused on middle and high school students in public schools in Riyadh, Saudi Arabia. To ensure a representative sample, the researchers utilized a three-stage sampling method during the initial quarter of the academic year. This approach, as noted by Greenfield and Greener (2016), enhances study efficiency and result accuracy. The selection process began by choosing a sample of schools from the Ministry of Education’s database, which encompasses a total of 877 schools with 236,909 students across public and private schools (Ministry of Education, 2022). Riyadh was divided into four geographical regions, with a regular random sample of schools selected from each region for survey implementation. Subsequently, a random sample of students within each chosen school was surveyed. The final sample for this study included 671 students randomly selected from 49 schools in Riyadh. Participants were distributed across different academic levels as follows: 122 (18.2%) sixth graders, 295 (44.0%) ninth graders, and 254 (37.9%) twelfth graders. This distribution ensured a diverse representation of students at varying stages of their educational journey. By incorporating students from these three academic levels, the study captured a broad spectrum of experiences and perspectives, enriching the overall representation of middle and high school students in Riyadh’s public schools. The use of a representative sample and random sampling method strengthens the external validity of the study’s findings, allowing for generalization of the results to the population of middle and high school students in Riyadh. The informed consent process and emphasis on no right or wrong answers helped to ensure the ethical conduct of the study. Overall, the sampling method used in this study appears to be appropriate for achieving the study’s research objectives. To gather data, an online survey was conducted. Web-based surveys have been recognized as valuable tools, utilizing the internet’s connectivity to reach individuals and organizations that may be challenging to engage through traditional means (Wright, 2005). By leveraging the web-based surveys, the study aimed to efficiently gather a sufficient sample size while overcoming potential challenges related to participant engagement and geographical limitations.

### 2.3 Measure

Given that self-efficacy is known to be domain-specific (Bandura, 1997; Pajares and Miller, 1995), the S-STEM survey was chosen for the current study. It was developed by the Friday Institute for Educational Innovation (2012) to measure changes in students’ confidence and efficacy in STEM subjects and their interest in STEM careers. The survey has been used in many previous studies (Bartholomew and Santana, 2021; Ching et al., 2019; Gremillion et al., 2019; Han et al., 2021; Popa and Ciascai, 2017; Razali et al., 2018; Zhang et al., 2021; Zhou et al., 2021). The survey comprises 26 items categorized into three distinct sub-constructs crucial for assessing STEM self-efficacy and career aspirations among participants: STEM Self-Efficacy, encompassing Science, Mathematics, Technology, and Engineering domains to evaluate confidence in related skills; Career Aspirations, probing interest and intentions in pursuing STEM careers; and Scales and Constructs, utilizing Likert scales to measure specific aspects of self-efficacy and aspirations toward STEM fields. Responses to attitude items are measured on a 5-point Likert-type scale, while responses to career interest items are measured on a 4-point scale. To adapt the survey to the Arabic language, two translators were involved in translating the survey from English to Arabic, and the translated survey was reviewed and edited by individuals with humanities and education backgrounds to ensure ease of comprehension. The Arabic version was then translated back to English by a different translator to ensure accuracy.

### 2.4 Validity and reliability

Content validity refers to the extent to which an instrument adequately encompasses all the relevant materials associated with the variable it intends to measure (Heale and Twycross, 2015). The researchers offered the survey online via Survey Monkey and conducted a pilot test with 10 students aged between 11 and 17 years old to ensure the survey’s clarity and avoid confusing questions. Feedback was collected, and a final copy was reviewed by a statistician to ensure alignment with research questions.

Table 1 provides evidence for the construct validity of the measurement instrument used to assess STEM self-efficacy. Construct validity refers to the extent to which a measurement accurately captures the underlying construct it intends to measure. The significant positive correlations between the majority of the items and the total score suggest that these items are measuring the intended construct effectively. Furthermore, the pattern of correlations across the items reinforces the construct validity of the measurement instrument. Items that are theoretically related to self-efficacy in STEM, such as items 9, 10, 12, 13, 18, 19, 20, and 28, exhibit high positive correlations with the total score. This consistency supports the notion that these items are aligning with the underlying construct of self-efficacy in STEM.

**Table 1:** Pearson correlation between each item of STEM self-efficacy with total score.

No.	The correlation coefficient	No.	The correlation coefficient	No.	The correlation coefficient
6	0.5345**	15	0.5125**	24	0.5700**
7	0.4372**	16	0.5180**	25	0.4174**
8	0.6008**	17	0.5669**	26	0.5871**
9	0.7318**	18	0.4867**	27	0.6681**
10	0.6024**	19	0.6138**	28	0.7440**
11	0.3552*	20	0.5622**	29	0.5759**
12	0.5484**	21	0.4156**	30	0.6392**
13	0.5957**	22	0.5379**	31	0.5728**
14	0.4699**	23	0.7128**		

** Correlation is significant at the 0.01 level (2-tailed).

*Correlation is significant at the 0.05 level (2-tailed).

Table 2 presents the Pearson correlation coefficients between each item of Career Interest and the total score. Analyzing the table, it is evident that the majority of the items exhibit significant positive correlations with the total score, indicating a strong relationship between these items and STEM career interest. Notably, several items stand out with particularly high correlation coefficients, such as items 32, 34, 41, and 42. These items demonstrate strong positive relationships with the total score, suggesting that individuals who express interest in these specific areas of STEM also exhibit higher overall STEM career interest. These findings provide support for the construct validity of these items in measuring STEM career interest accurately.

**Table 2:** Pearson correlation between each item of career interest with total score.

No.	The correlation coefficient	No.	The correlation coefficient	No.	The correlation coefficient
32	0.7481**	36	0.7184**	40	0.5991**
33	0.7034**	37	0.4113**	41	0.8376**
34	0.7903**	38	0.7651**	42	0.7888**
35	0.6623**	39	0.3746**	43	0.5854**

**Correlation is significant at the 0.01 level (2-tailed).

Table 3 shows that the Cronbach’s Alpha for this study ranged from 0.85 to 0.91, indicating that the survey items were reliable. A score of 0.70 or higher is generally considered acceptable for establishing reliability (Lance et al., 2006). Overall, the S-STEM survey is a well-established and widely used instrument for measuring students’ attitudes toward STEM and their interest in STEM careers with high Cronbach’s Alpha values suggest that the survey items were reliable for use in this study.

**Table 3:** Cronbach’s Alpha for the reliability of the factors of STEM self efficacy.

Factor	No. of items	Alpha
Self-efficacy toward Mathematics	8	0.86
Self-efficacy toward Sciences	9	0.85
Self-efficacy toward Engineering and Technology	9	0.88
The part one of the questionnaire (Self-efficacy toward (STEM))	26	0.91
The part two of the questionnaire (the career interest)	12	0.89

### 2.5 Ethics

Data collection was conducted following the approval of Princess Nourah bint Abdulrahman University (PNU), IRB Registration Number with KACST, KSA: HAP-01-R-059.

## 3 Data analysis

### 3.1 STEM career interest

Table 4 provides a breakdown of the distribution of responses based on mean scores and categorizes them into four levels of interest. The “Very interested” category represents mean scores ranging from 3.26 to 4.00. The “Interested” category includes mean scores ranging from 2.51 to 3.25. The “Not interested” category encompasses mean scores ranging from 1.76 to 2.50, while the “Not interested at all” category includes mean scores ranging from 1.00 to 1.75.

**Table 4:** Frequencies, percentages, means, and standard deviations of the sample STEM career interest.

List of the careers		Very interested	Interested	Not interested	Not interested at all	Mean	Std. deviation	Rank
Physics	Freq	88	274	228	81	2.55	0.87	6
	%	13.1	40.8	34	12.1			
Environmental work	Freq	63	250	256	102	2.41	0.86	9
	%	9.4	37.3	38.1	15.2			
Biology and zoology	Freq	115	241	211	104	2.55	0.95	6
	%	17.1	35.9	31.5	15.5			
Veterinary work	Freq	114	184	237	136	2.41	0.99	9
	%	17	27.4	35.3	20.3			
Mathematics	Freq	116	253	209	93	2.58	0.93	5
	%	17.3	37.7	31.1	13.9			
Medicine	Freq	316	210	87	58	3.17	0.96	1
	%	47.1	31.3	13	8.6			
Earth science	Freq	151	226	206	88	2.66	0.97	4
	%	22.5	33.7	30.7	13.1			
Computer science	Freq	175	254	162	80	2.78	0.97	3
	%	26.1	37.9	24.1	11.9			
Medical science	Freq	210	245	158	58	2.90	0.94	2
	%	31.3	36.5	23.6	8.6			
Chemistry	Freq	118	203	249	101	2.50	0.95	7
	%	17.6	30.2	37.1	15.1			
Energy	Freq	87	182	262	140	2.32	0.95	10
	%	13	27.1	39	20.9			
Engineering	Freq	104	204	245	118	2.44	0.95	8
	%	15.5	30.4	36.5	17.6			
Mean for total						2.61	0.57	

In Table 4, the mean scores range from 2.32 to 3.17. The table allows for a clear comparison of the level of interest across different careers. It can be observed that Medicine has the highest overall interest with a frequency of 526 and a percentage of 78.4. Computer Science and Medical Science also received relatively high levels of overall interest, ranking second and third, respectively. On the other hand, Energy (2.32), Environmental Work (2.41), and Veterinary Work (2.41) received lower mean scores for overall interest, indicating comparatively lower levels of interest in these fields. The mean for the total responses is 2.61, which represents the average overall interest across all careers. Overall, the table provides insights into the level of interest expressed by the respondents for different careers, allowing for a comparison based on mean scores. The mean values offer an indication of the average interest level, while the standard deviation would provide further context regarding the spread of responses for each career.

### 3.2 STEM self-efficacy

S-STEM questionnaire was utilized for analysis. Items were based on a five-item Likert scale, consisting of the following categories: “Very much agree,” “Agree,” “Neutral,” “Disagree,” and “Very much disagree.” The range calculated for the scale was 5–1 = 4. Dividing this range by the number of categories (5) resulted in 4/5 = 0.80, which was the length of each category on the five-point scale. Finally, this length (0.80) was added to the lowest grade of the scale, which was 1. Consequently, the first category was calculated to range from 1 to 1.80. For the subsequent category, 0.80 was added to the previous category’s upper limit, and this process was repeated for the remaining categories. Table 5 displays the results of the analysis, indicating that the mean self-efficacy for female students in STEM in Riyadh, Saudi Arabia, is 3.7.

**Table 5:** Mean’s and std. deviation for total of the self-efficacy Saudi female student toward STEM.

The factor	Mean*	Std. deviation	Rank
Self-efficacy toward Mathematics	3.70	0.78	2
Self-efficacy toward Sciences	3.74	0.72	1
Self-efficacy toward Engineering and Technology	3.66	0.79	3
The total of self-efficacy toward (STEM)	3.70	0.56	-

^*^The mean of 5 degrees.

### 3.3 The correlation between career interest and STEM self-efficacy

In the analysis of Table 6, hypothesis testing was conducted to determine the significance of the correlations presented for different STEM careers across the fields of STEM. The null hypothesis assumes that there is no significant correlation between the STEM careers and the corresponding fields STEM. In other words, the correlation coefficients are equal to zero. The alternative hypothesis proposes that there is a significant correlation between the STEM careers and the respective fields. It suggests that the correlation coefficients are not equal to zero, indicating a positive relationship between the variables. By conducting the hypothesis testing using the Spearman correlation coefficient test at a significance level of 0.01, the study aimed to determine whether the observed correlations between the STEM careers and the fields of STEM were statistically significant and not due to random chance.

**Table 6:** Spearman correlation coefficients to measure the relationship between career interest and STEM self-efficacy.

STEM	Science self-efficacy			Engineering and technology self-efficacy			Mathematics self-efficacy		
Career Interest	Correlation	Sig.	Description	correlation	Sig.	Description	Correlation	Sig.	Description
Physics	0.24	0.00	Direct (Positive)	0.37	0.00	Direct (Positive)	0.14	0.00	Direct (Positive)
Environmental work	0.25	0.00	Direct (Positive)	0.27	0.00	Direct (Positive)	0.11	0.005	Direct (Positive)
Biology and zoology	0.31	0.00	Direct (Positive)	0.22	0.00	Direct (Positive)	0.07	0.069	Almost non-existent
Veterinary work	0.15	0.00	Direct (Positive)	0.16	0.00	Direct (Positive)	0.05	0.193	Almost non-existent
Mathematics	0.18	0.00	Direct (Positive)	0.39	0.00	Direct (Positive)	0.42	0.00	Direct (Positive)
Medicine	0.33	0.00	Direct (Positive)	0.12	0.003	Direct (Positive)	0.16	0.00	Direct (Positive)
Earth science	0.28	0.00	Direct (Positive)	0.30	0.00	Direct (Positive)	0.02	0.564	Almost non-existent
Computer science	0.10	0.011	Direct (Positive)	0.38	0.00	Direct (Positive)	0.17	0.00	Direct (Positive)
Medical science	0.37	0.00	Direct (Positive)	0.24	0.00	Direct (Positive)	0.15	0.00	Direct (Positive)
Chemistry	0.31	0.00	Direct (Positive)	0.34	0.00	Direct (Positive)	0.23	0.00	Direct (Positive)
Energy	0.26	0.00	Direct (Positive)	0.40	0.00	Direct (Positive)	0.16	0.00	Direct (Positive)
Engineering	0.21	0.00	Direct (Positive)	0.49	0.00	Direct (Positive)	0.29	0.00	Direct (Positive)

The results presented in the Table 6 show that there is a positive and statistically significant correlation between the students’ interest in different STEM careers and their self-efficacy toward STEM. The positive correlation coefficients indicate that the higher self-efficacy toward STEM, the greater the female students’ interest in a particular STEM field, this suggests that higher STEM Self-Efficacy STEM Self-Efficacy can increase interest in STEM fields, as students may feel more confident in their ability to pursue and succeed in these fields. The direct positive relationship between interest in various STEM careers and STEM Self-Efficacy is consistent across all fields, However, it is interesting to note that there is almost no relationship between the interest of the sample in Veterinary, Work and Earth Science, and Biology and Zoology, and their self-efficacy toward Mathematics.

## 4 Discussion

### 4.1 STEM career interest

The current study demonstrates a high level of interest in medicine, with 47.1% of students reporting being “Very interested,” is not surprising, as medicine is a highly respected and well-paid profession that offers various opportunities for career advancement. In their meta-analysis of the gender gap in STEM education, Wang and Degol (2016) found that females have dominant representation in the medical and health sciences sectors, yet they remain underrepresented in other STEM fields, especially those that require intensive mathematical skills. They attributed the underrepresentation of females in STEM fields to factors such as cognitive abilities, occupational preferences, work-family balance, field-specific ability beliefs, gender-related stereotyping, and workplace bias.

The lower interest levels observed in physics and environmental work careers, as presented in Table 4, may be attributed to several factors. One possible explanation is a lack of exposure to these fields or a limited understanding of the career opportunities they offer. This aligns with previous research findings by Su et al. (2009), which indicate that women generally exhibit greater interest in careers involving human interaction, while men tend to be more interested in careers involving object-oriented work. Given that many STEM careers predominantly involve object-oriented work, this gender disparity in career preferences may contribute to the underrepresentation of women in STEM fields. Moreover, the World Economic Forum (WEF) in 2020 has also acknowledged the marginalization of females in STEM domains. Considering these factors, it becomes evident that there are complex dynamics at play influencing individuals’ interest and career choices in STEM fields, particularly in physics and environmental work. Further reflection and analysis on these findings can provide valuable insights into addressing the underrepresentation of certain groups in STEM careers and fostering greater diversity and inclusivity within these fields.

### 4.2 STEM self-efficacy

The results indicate that female students in Riyadh, Saudi Arabia, have a higher mean self-efficacy in STEM (3.7) compared to female students in Southeastern Arizona, USA (3.16) reported by Falco and Summers (2017). This finding also surpasses the scores of female students in China (3.53), as reported by Zhou et al. (2021). The researcher suggests that the positive self-efficacy levels among female students in STEM fields may be attributed to the school environment, where they have regular interactions with and exposure to female teachers who serve as role models in mathematics, science, and information technology. This aligns with previous research by Stout et al. (2011), which emphasized the significant influence of female STEM lecturers on fostering positive self-efficacy among female students. The gender-segregated nature of local schools in Saudi Arabia may contribute to the higher self-efficacy observed among female students. This environment allows girls to develop and excel without gender-based comparisons or traditional gender role influences. From a liberal feminism perspective, Saudi schools are gender-segregated, providing equal opportunities for both male and female students. The curriculum taught in public schools is identical for both genders, allowing female students to develop and grow in an environment without disparities. This approach reflects efforts to promote gender equality and provide equal educational opportunities for all students in Saudi Arabia. Stout et al. (2011) found that exposure to same-sex influential experts in the field of engineering increased female student’s positive attitudes toward STEM and resulted in aspiration to pursue STEM careers.

However, further studies involving both genders are needed to validate and gain a more comprehensive understanding of these initial findings. The findings can also be attributed to the efforts made by Saudi Arabia to promote gender equality in STEM education and professions (Aljuaid and Liu, 2023). Saudi Arabia has set an ambitious target to increase the percentage of women in the workforce from 22 to 30% by 2030, with a specific focus on boosting women’s participation in STEM careers. Gender and STEM fields in Saudi Arabia have been viewed as distinct realms, offering limited prospects for women to engage in STEM professions. Although the Saudi government has endorsed educational equality, resulting in increased female participation in the workforce and educational accomplishments.

### 4.3 Career interest and STEM self-efficacy

The data shows a positive and direct relationship between STEM self-efficacy and interest in careers such as Physics, Environmental Work, Biology and Zoology, Mathematics, Medicine, Computer Science, Medical Science, Chemistry, Energy, and Engineering. However, there seems to be negligible correlation between the sample’s interest in Veterinary Work, Earth Science, and Biology and Zoology and their self-efficacy specifically in mathematics.

The review of existing literature underscores the importance of student attitudes and STEM self-efficacy in predicting their inclination toward pursuing careers in STEM fields. Numerous studies have illustrated how attitudes and interests significantly influence students’ career decisions, particularly in subjects like science and mathematics, shaping their interest in STEM careers across various educational stages (Gremillion et al., 2019; Razali et al., 2018). Research has delved into the role of self-efficacy in STEM and its impact on career choices, taking into account variables such as gender and school types. An extensive survey conducted by Wiebe et al. (2013) involving 15,000 students explored the interplay between STEM careers, attitudes toward STEM subjects, and interest in STEM careers at different academic levels. The findings revealed that students generally hold moderately positive attitudes toward STEM. However, female students exhibited lower levels of interest in specific STEM career paths compared to their male peers, who demonstrated comparable interest levels across all STEM disciplines. In a study by Chan (2022), the relationship between career interest and STEM interest was investigated, uncovering a positive correlation between the two variables. Additionally, a stronger sense of self-efficacy was associated with heightened interest in pursuing careers in STEM fields.

The positive direct relationship between STEM self-efficacy and STEM career interest in female students do differ from findings in some previous studies. For instance, Wiebe et al. (2018) and Faber et al. (2013) reported lower interest in core STEM careers among female students. However, a study by Abdulwahed et al. (2013) in Qatar, a neighboring Gulf country, discovered that women exhibit more interest in STEM professions compared to men, emphasizing the potential impact of culture and educational settings. Moreover, Fadlelmula et al. (2022) suggested that the selection of STEM fields for study and careers is influenced by various factors both within and outside the classroom, including traditions, cultural norms, parental support, and the incorporation of STEM in the curriculum (Fadlelmula et al., 2022). Research conducted in The Netherlands identified a lack of female role models in STEM and insufficient efforts to inspire girls to pursue STEM studies as reasons for the low interest in STEM careers among female students (Van Wassenaer et al., 2023). Studies have also indicated that stereotypes surrounding science and mathematics can discourage adolescent girls from entering STEM careers (Cundiff et al., 2013; Lane et al., 2011).

Notably, Fadlelmula et al. (2022) highlighted that while interest in STEM fields has been extensively studied in Western countries and other regions, it remains relatively understudied in the Arab world. Pasha-Zaidi and Afari (2016) conducted a study in the UAE exploring the impact of gender on students’ perceptions of mathematics and science instructors at the university level, revealing a distinct influence of gender on how students perceive their instructors. They recommended further research on gender preferences in the Gulf region to better address student recruitment in STEM disciplines.

What calls for investigation is that despite the positive relationship between STEM self-efficacy and STEM career interest found in the current research, there is a low number of female employees in STEM fields in Saudi Arabia. Islam M. M. (2019) and Islam S. I. (2019) has highlighted a significant imbalance of Saudi female STEM graduates compared to women in the labor force in STEM fields. Culture is a significant factor that may impede female progress in STEM fields in the Gulf Cooperation Council (GCC) area, where traditional gender roles may limit female opportunities for employment to domestic jobs, teaching, and healthcare (Al-Alawi et al., 2019).

Furthermore, the positive direct relationship between the interest of the sample in STEM careers and their self-efficacy toward science raises the question of the influence of gender and the need to compare female and male students to determine if this trend is related only to female students or if it also applies to male students in Saudi Arabia. Further research is needed to explore the gender differences and also the role of socioeconomic factors in influencing STEM career interest and self-efficacy. This can help to identify any potential barriers to STEM participation and inform the development of targeted interventions to promote equitable access to STEM education and careers for all students. Saudi Arabia has seen a significant gender gap in mathematics performance. According to the Trends in International Mathematics and Science Study Test (TIMSS), Saudi Arabia ranked second among countries with a notable gender gap in 4th-grade mathematics, with girls scoring 26 points higher than boys (ETEC, 2019). This gender gap is among the highest observed in TIMSS and the Program for International Student Assessment (PISA), with girls consistently outperforming boys in Saudi Arabia. While in most OECD countries, boys tend to outperform girls in mathematics, the situation is reversed in Saudi Arabia, where girls achieve higher grades in this domain. Similarly, in science, Saudi girls outperformed boys by 29 points in PISA (2019), while the average gender gap across OECD countries was only two points, with girls outperforming boys (OECD, 2019).

Regarding lack of interest in Earth Science careers and Veterinary Work among girls in Riyadh, researchers hypothesize that the absence of a dedicated Earth Science curriculum at the intermediate level in the educational system may contribute to the reduced interest and lower self-efficacy in this field. The omission of a tailored Earth Science program could hinder the sample’s exposure and understanding of the subject. Additionally, the limited enthusiasm observed for Veterinary Work within the select sample from Riyadh, a metropolitan city distant from agricultural or farming fields, is proposed as a contributing factor. The absence of direct exposure to agricultural or rural settings, where veterinary work is commonly associated, may explain the diminished interest and limited self-efficacy in pursuing a career in this field. This study sheds light on the importance of tailored educational programs and exposure to relevant environments to foster interest and self-efficacy in Earth Science and Veterinary Work among girls, ultimately informing strategies to promote career diversity and engagement in these fields.

Based on the findings of the research, it is recommended that mentorship programs, networking opportunities, and job fairs be established to increase the visibility of successful female STEM professionals. These initiatives can provide guidance, support, and skill development opportunities to female students and professionals in STEM, which can encourage enrollment in these fields. Role models and mentorship opportunities have been found to be crucial factors that can significantly enhance self-efficacy beliefs among women (Stout et al., 2011). These programs can also help to address the lack of female representation in STEM fields and promote gender equality and diversity in the workforce. Also, the observed gender gaps in STEM interest and self-efficacy suggest a need for targeted interventions to encourage female participation in traditionally male-dominated fields. The study highlights the impact of cultural norms on career choices, particularly in the Gulf region. Addressing traditional gender roles and promoting inclusivity in STEM fields is crucial for fostering a diverse and skilled workforce.

### 4.4 Limitations and future research agenda

This study has provided valuable insights into STEM career interest and STEM self-efficacy among girls. However, it is important to acknowledge the limitations of this research, which focused solely on girls in a metropolitan city. As such, the findings may not be generalizable to other regions or rural areas. To address these limitations and further enhance our understanding of STEM self-efficacy among girls, several avenues for future research can be explored.

Firstly, conducting studies in rural cities and other metropolitan areas can provide a more comprehensive perspective on STEM self-efficacy among girls. This would allow for a broader representation of girls from different backgrounds and contexts, helping to identify any regional differences or similarities in their attitudes toward STEM. By including girls from diverse regions, we can gain a deeper understanding of the challenges and opportunities they face, and develop targeted interventions to promote STEM self-efficacy and interest.

Furthermore, future research can extend beyond the borders of Saudi Arabia to explore the factors that influence STEM self-efficacy among girls in different regions of the world. This comparative approach can shed light on the cultural, societal, and educational factors that contribute to or hinder girls’ engagement with STEM fields. Understanding these factors will enable the development of tailored strategies and interventions that are effective in different contexts.

Additionally, it is important to investigate the relationship between STEM career interest, self-efficacy, and gender differences. Comparative research can explore whether the positive relationship between interest in STEM careers and self-efficacy is specific to female students or if similar patterns exist among male students as well. Such research can provide insights into the unique challenges faced by girls and boys in STEM education and help develop inclusive approaches to promote equitable access and participation in STEM fields.

Moreover, considering the influence of socioeconomic factors on STEM self-efficacy is crucial. Research that examines the intersectionality of gender and socioeconomic status can uncover barriers and disparities in STEM education and careers. This knowledge can inform policies and interventions aimed at ensuring equitable opportunities for all students.

Finally, it is important to recognize that self-efficacy is not a fixed trait and can be developed through various sources of support, such as instructors, parents, mentors, counselors, and peers. Future research can explore the impact of these support systems on the development of STEM self-efficacy among girls and identify effective strategies for fostering self-efficacy through targeted interventions and mentorship programs.

## 5 Conclusion

Overall, the current study has shed light on the relationship between STEM career interest and STEM self-efficacy among female students in Riyadh, Saudi Arabia. The findings suggest that there is a positive and direct relationship between STEM self-efficacy and interest in STEM careers such as Physics, Environmental Work, Biology and Zoology, Mathematics, Medicine, Computer Science, Medical Science, Chemistry, Energy, and Engineering. However, the lack of interest in Veterinary Work and Earth Science careers may be attributed to limited exposure to these fields in the educational system and cultural factors. While this study has provided valuable insights, further research is needed to overcome the limitations and expand our understanding of STEM self-efficacy among girls. By conducting studies in diverse regions, exploring gender differences, considering socioeconomic factors, and investigating the impact of support systems, we can develop a comprehensive understanding of STEM self-efficacy and contribute to the development of effective policies and programs that promote female participation in STEM fields.

## Data Availability

The original contributions presented in the study are included in the article/supplementary material, further inquiries can be directed to the corresponding author.
